# Assessing communicative-pragmatic ability in telehealth: e-ABaCo in autistic individuals

**DOI:** 10.3389/fpsyt.2025.1568108

**Published:** 2025-04-28

**Authors:** Ilaria Traetta, Ilaria Gabbatore, Alessandra Aimar, Giuseppe Maurizio Arduino, Francesca M. Bosco

**Affiliations:** ^1^ Department of Psychology, Research Group on Inferential Processes in Social Interaction (GIPSI) Research Group, University of Turin, Turin, Italy; ^2^ Department of Humanities, Research Group on Inferential Processes in Social Interaction (GIPSI) Research Group, University of Turin, Turin, Italy; ^3^ Centro Riabilitazione Ferrero, Alba, Italy; ^4^ Centro Autismo e Sindrome di Asperger ASL CN1, Mondovì, Italy

**Keywords:** autism spectrum disorders, autistic people, adolescents, pragmatics, telehealth, tele-evaluation, tele-assessment, remote assessment

## Abstract

**Introduction:**

Autism Spectrum Disorder (ASD) is a neurodevelopmental disorder characterized by difficulties in social communication. Autistic individuals who are verbally fluent, often show difficulties in pragmatic ability, i.e. the capacity to use language and other expressive means, as gestures or the tone of the voice, to convey meaning in a given context. During the COVID-19 pandemic, the use of various technologies such as videoconferencing and internet for the delivery of healthcare services, i.e. telehealth, has proven to be effective, accessible and safe tools for remote healthcare. However, there are few tools to assess pragmatic skills in telehealth. This study investigates the effectiveness of the telehealth-adapted Assessment Battery for Communication (e-ABaCo), a clinical tool previously validated for in-person assessments, to evaluate pragmatic abilities in autistic individuals. We expect a substantial equivalence in performance for the administration of the adapted version of e-ABaCo compared to the face-to-face one.

**Methods:**

We compared the performance of 30 autistic adolescents, of which 15 assessed via telehealth (ASD TH) and 15 assessed face-to-face (ASD FtF), with that of 15 adolescents with typical development (face-to-face assessment). The groups were matched for age, sex, and intellectual quotient. E-ABaCo was used to assess both comprehension and production of communicative ability realized through different expressive means, i.e. linguistic, extralinguistic and paralinguistic, as well as social appropriateness.

**Results:**

In line with the expectation, the pragmatic performance of autistic adolescents showed a substantial equivalence when comparing the assessments conducted via telehealth and face-to-face modality. Moreover, in line with the relevant literature, there was a significant difference between the ASD groups’ performance (both FtF and TH) and the control group of the adolescents with typical development (CG FtF) in all pragmatic aspects assessed, i.e. the Pragmatic total score, comprehension and production abilities, and all the expressive means investigated.

**Discussion:**

These results confirm the potential usefulness of telehealth assessment procedures, and demonstrate the sensitivity and validity of e-ABaCo for conducting an effective assessment of pragmatic skills in on-line modality in autistic adolescents.

## 1 Introduction

Communication is a vital aspect of human life. It enables individuals to express ideas, share knowledge, convey emotions and create meaning in social interactions. This complex ability goes beyond language and includes additional cues such as prosody — tone of voice, intonation and rhythm — as well as extra-linguistic elements such as facial expressions and gestures (1). The ability to use language and other expressive forms appropriately in different contexts is referred to as pragmatics (2, 3). Developing effective and context-appropriate communication skills is crucial for successfully managing everyday interactions.

Autism spectrum disorder (ASD) is a lifelong neurodevelopmental disorder characterized by difficulties in social communication (DSM-5-TR; 4). Current evidence suggests that autistic traits exist along a continuum, with difficulties in social communication ranging from mild to severe. These difficulties may vary in these individuals: they may include language processing challenges (e.g. syntax and vocabulary) or involve more sophisticated communicative skills such as pragmatic ability (5–7). Even when linguistic skills are intact, autistic people^1^ may face challenges in various aspects of communication (10–13). These difficulties may include interpreting and using paralinguistic cues – such as rhythm, intonation and prosody – that accompany speech (14, 15). Paralinguistic cues serve as essential expressive mean to convey intentions and emotions. For autistic people, these aspects can present a particular challenge, both in coding the others’ facial expressions and in expressing their own. This cognitive effort can be mentally exhausting and often leads to fatigue during social interactions, which can hinder the development and maintenance of interpersonal relationships (13, 16, 17). Pragmatic ability also includes the integration of language and social context to effectively interpret intended meaning and fill the gap between the speakers’ literal meanings and their actual intention. A growing number of studies highlighted the systematic difficulties of individuals in the autistic spectrum in non-literal language processing, as testified by their tendency to literally interpret figurative expressions (18, 19), such as indirect speech acts (20), irony (21) and metaphors (22). Moreover, people on the spectrum may have difficulties with conversational skills, such as understanding when and how to start a conversation, maintaining a topic and taking turns appropriately or modulating the conversation in a way that is appropriate to the context (23). Moreover, autistic people may show difficulties in communicative interaction when required to handle non-verbal/extra-linguistic aspects, such as gestures and body movements (21). The use of non-verbal expressive means is crucial in situations where verbal communication is limited or not possible. For example, imagine communicating in a noisy environment or trying to convey an urgent message to someone who is busy with something else, e.g. on the phone. In addition, non-verbal aspects often complement verbal communication by improving the precision and clarity of the intended message, e.g. when explaining complex concepts. Often autistic people accompany their speech with few gestures or do not integrate it with gestures and speech (24).

Several previous studies have examined the social and communication skills of autistic children (e.g. 25, 26), but there are comparatively few studies focusing on these skills at later stages of development (10, 11, 27, 28). However, difficulties in pragmatic communication are not limited to preschool and school age, but persist through adolescence and early adulthood (29), period in which pragmatic ability continues to improve until it reaches a plateau at around 25-30 years of age (30). A similar pattern was observed by Bischetti et al. (31), who reported that pragmatic abilities peak between 30 and 45 years and decline after 45 years, following a developmental trajectory consistent with changes in other cognitive domains across the lifespan.

Adolescence is a critical developmental period characterized by important cognitive developments, supported by remarkable structural and functional changes in the brain, particularly in social cognition (e.g., (32, 33) and executive functions (e.g., 34). During this period, more advanced communication skills than in childhood are needed to prepare individuals for the challenges of adult life. Adolescent develop the ability to think abstractly and begin to use abstract vocabulary (e.g., metacognitive terms) and advanced syntactic structures, and they intensify the use of figurative language. These advances facilitate the understanding of non-literal meanings, such as figurative or idiomatic expressions, and enable contextually appropriate interpretations (35). During this phase, communication also becomes more sophisticated due to the increasing demand in social life. Adolescents typically intensify their engagement with the external world, e.g. school teachers, employers, romantic or sexual partners; moreover, peer influence becomes more important and sensitivity to social exclusion grows (36). Despite this heightened vulnerability, however, adolescents retain the ability to rely on social stereotypes to navigate social interactions, which may serve as a compensatory mechanism for understanding social norms and expectations (37).

The lack of studies investigating pragmatic ability in adolescence, even despite its relevance for social interactions, is related to the scarcity of validated tools for the pragmatic assessment that may be sensitive to investigate this ability during this age period. Indeed, the majority of pragmatic tools used to investigate communicative ability in ASD in the face-to-face modality, have been developed for preschool and school-age children. They include the Pragmatic Language Skills Inventory (PLSI, 38), the Children’s Communication Checklist (CCC–2, 39), the Pragmatic Protocol (40), the Test of Pragmatic Language (TOPL-2, 41) and the Pragma Test (42, 43). However, there are currently few standardized tools specifically designed to assess pragmatic skills in adolescence (44): one is the Assessment Battery for Communication (ABaCo, 45, 46) and the other is Pragmatic Abilities and Cognitive Substrates (APACS, 47). While the APACS was, so far, specifically (48) used to assess pragmatic abilities in Hebrew-speaking adolescents, the ABaCo has been already used with Italian-speaking autistic adolescents (21, 49), and this is the reason why we decided to adapt this tool for the use in telehealth.

Quite a novelty in the most recent development of the clinical practice is the possibility to conduct remote or on-line assessment (50, 51). The restricted access to assessment and treatment services experienced during the COVID-19 pandemic, led to a clear shift towards alternative methods such as telehealth. This shift was particularly critical as the pandemic further widened the gap between professionals and patients, which had an even greater impact on people with disabilities. Among the frail populations, autistic people were particularly vulnerable during the pandemic due to the difficulties in communication and socialization associated with the diagnosis (4). Predictability, a key component of autistic people’s wellbeing, i.e. routine, was profoundly disrupted, exacerbating difficulties in many areas - including access to healthcare services (52). During the COVID-19 pandemic, the disruption to services caused by staff turnover, resource shortages, the mandatory closure of facilities and reduced or altered access to essential specialist support has had a profound impact not only on autistic people, but also on their families, caregivers, natural support networks and the professionals who take care of them. In this period, anxiety and depression were the most common consequences among young people. In particular, the data indicated that older adolescents exhibited more depressive symptoms during lockdown than their younger peers, likely due to their greater need for social contact and interpersonal relationships (53). Even before the pandemic, research showed that autistic adolescents and young adults were four times more likely to visit emergency departments compared to their typically developing peers (54). In addition, many autistic people did not have access to sufficiently powerful devices or software to download or use digital materials (55), which exacerbated their challenges during such times of disruption.

To mitigate the negative consequences of the COVID-19 pandemic, the introduction of digital technologies has been accelerated and the rapid integration of telehealth has been promoted in various areas (56). Telehealth, also known as telepractice, telemedicine, telecare, telepsychiatry, refers to the use of various technologies such as video conferencing and the internet to deliver healthcare services (57). Telehealth is not a new concept. There is already some evidence that it is a practical alternative or integration to traditional face-to-face assessments (50), with high levels of client satisfaction (58). The benefits are manifold: telehealth is characterized by security, cost and time efficiency and allows professionals (e.g., therapists, psychologists) and researchers to engage with patients or participants in real time, overcoming geographical barriers (59). This approach is particularly beneficial when a face-to-face interaction is not possible or difficult to manage, as for people living in remote or rural areas (60, 61). In research setting, telehealth also facilitates the inclusion of geographically diverse groups of participants, overcoming the limitations of traditional methods that often rely on participants from a single location (62).

In the field of psychological and neuropsychological tele-assessments, there is modest but growing evidence for the validity, reliability and effectiveness of remote methods with generally positive results (50, 51). Studies have shown equivalence between telehealth and face-to-face modalities for clinical interviews, self-report questionnaires, neuropsychological testing and language assessments (50, 51, 59, 63–65).

Specifically for ASD, various services have been delivered via telehealth, such as diagnostic assessments, early intervention programs, family consultation and language therapy (66). For diagnostic purposes, some instruments such as the standardized Autism Diagnostic Interview-Revised (ADI-R) and the Autism Diagnostic Observation Schedule (ADOS) Module 1 activities (ADOS, ADI-R; 67) or newer instruments such as the TELE-ASD-PEDS, a caregiver-mediated remote observation assessment, have also been successfully adapted for use in telehealth (68), even if more research is needed to confirm their validity (69). Most of the early teleintervention programs are not direct to autistic individuals but are addressed to their parents, the such as the “ImPACT program”, a telehealth-mediated intervention for young autistic children (70, 71), or imitation training, a program meant for parents of autistic children with the aim to increase the spontaneous imitation skills (72). A study conducted by Boisvert et al. (73) investigated the effectiveness of a language intervention delivered via telehealth and showed an improvement in narrative skills with telehealth compared to the traditional face-to-face method in a single case study with an 11-year-old boy diagnosed with autism. A systematic review of Knutsen et al. (74) showed that the majority of participants reported high level of satisfaction using telehealth for monitoring ASD care at home and for school-based programs, with satisfaction levels similar to those of families receiving in-person services. Most of the studies reviewed in Knutsen et al. (74) were pilot or preliminary studies and examined the feasibility and effectiveness of telehealth in education, treatment, and diagnosis of ASD. These studies proved telehealth effective for delivering behavioral interventions and coaching parents, teachers, and professionals. Additionally, 12 case reports and small single-case designs showed that telemedicine was effective in both diagnosis and treatment, with 5 studies that effectively conduct tele-assessment in autistic children (75–77), adolescents (78) and adults (79). Despite some technological and logistical challenges, these findings support the use of telemedicine to assist individuals in the autism spectrum, especially for those without access to local specialists.

Despite evidence for the feasibility and statistical reliability of telehealth assessment of psychological and neuropsychological assessments via remote diagnosis and traditional face-to-face assessment, studies specifically assessing pragmatic communication skills in autistic adolescents have not yet been adequately explored, representing a significant gap in the current literature which remains limited to a little number of studies (50, 74, 80, 81).

To the best of our knowledge, only one tool has been used in a telehealth setting for pragmatic assessment, specifically in Italian context, namely APACS Brief Remote (82). The APACS Brief Remote is a newly developed online tool based on the original test Assessment of Pragmatic Abilities and Cognitive Substrates (APACS, 47) a clinical instrument for the assessment of receptive and expressive pragmatic ability with a focus on discourse and non-literal meaning (e.g. metaphors, idioms). The work of Bischetti et al. (82) tested the psychometric properties of a new, shortened version of the original test aimed at the rapid remote assessment of pragmatic ability in Italian-speaking healthy adults. The analysis showed that this alternative short form is equivalent to the face-to-face test and has good psychometric properties, including reliability (internal consistency, test-retest and inter-rater) and validity. In addition, participants reported positive experiences with remote administration, which supports the feasibility of the test. However, this study focused on individuals aged 19 to 90 years, thus lacking data specific to adolescents, and is not tailored to the assessment of autistic individuals.

### 1.1 The study aims

The present study aims to describe the adaptation process and to evaluate the feasibility of the Assessment Battery for Communication (ABaCo; 45, 46) in telehealth (e-ABaCo) and to explore the effectiveness of e-ABaCo in providing a comprehensive assessment of the communicative-pragmatic ability of autistic adolescents in telehealth; our aim is to fill the current gap in the literature, where no specific communicative-pragmatic tools or comprehensive data are available for this specific population. To achieve this goal, we compared the performance of a group of 15 adolescents with typical development assessed face-to-face (CG FtF) with that of 30 autistic adolescents, divided into two groups: 15 autistic adolescents assessed via telehealth (ASD TH) and 15 autistic adolescents assessed face-to-face (ASD FtF). This approach allowed us to examine both the differences between autistic and typical development participants (CG FtF) and the impact of the assessment method (TH vs. FtF) on performance.

The ABaCo is a validated clinical instrument to assess several communicative aspects realized through different expressive modalities, i.e. linguistic, non-verbal/extralinguistic and paralinguistic, that has already demonstrated its effectiveness in assessing pragmatic abilities in ASD in the face-to-face modality (21, 49). ABaCo has been validated and normative data have been established for the Italian adult population (83). In addition, ABaCo has two equivalent forms (A and B) that have been validated (46) and used in different clinical contexts (49, 84, 85) Equivalent forms are particularly valuable because they substantially attenuate practice or familiarity effects that can affect the accuracy of change measures (e.g., by underestimating the progression of a deficit or overestimating treatment effects; see 86). Several studies have demonstrated the efficacy of the adapted version for children and adolescents of ABaCo in assessing pragmatic in typical development (87) and atypical development, such as autistic adolescents (21), children with Special Needs (88). Moreover, ABaCo has proven effective for the pragmatic assessment in several adult clinical populations such as people with acquired focal brain injury (89–92) and individuals with schizophrenia (93, 94). Some of the scales that make up ABaCo have been adapted for the English-speaking (95), Finnish (96), Serbian (97) and Portuguese cultural context (98).

The main aims of the study were:

- To adapt the Assessment Battery for Communication (46, 83) to be used for tele-assessment, i.e., e-ABaCo.
- To evaluate the effectiveness of e-ABaCo in describing the communicative-pragmatic profile of autistic adolescents.

First of all, we expect both ASD groups (i.e., telehealth and face-to-face) to have lower performance scores than the typical development group (CG FtF). Furthermore, we hypothesize that the performance of a group of autistic adolescents assessed via telehealth with e-ABaCo, will not differ from the performance of a comparable group assessed face-to-face with ABaCo.

## 2 Materials and methods

### 2.1 Participants

The sample consisted of 15 adolescents with typical development (CG FtF group) and 30 autistic adolescents. Specifically, the CG FtF group consisted of 15 adolescents with typical development (3 females; age between 12 and 18 years, *M* = 15.26, *SD* = 2.40; years of education attained: between 6 and 14 years, *M* = 9.93, *SD* = 2.63; Intellectual Quotient (IQ): between 85 and 113, *M* = 96.00, *SD* = 8.12). The telehealth ASD group (ASD TH) comprised 15 autistic adolescents (2 females; age between 12 and 18 years; *M* = 14.00, *SD* = 1.96; years of education attained: between 7 and 13 years, *M* = 8.93, *SD* = 1.75; IQ: between 66 and 124, *M* = 93.20, *SD* = 18.33. The face-to-face ASD group (ASD FtF) comprised 15 autistic adolescents (3 females; age between 12 and 18 years, *M* = 14.36, *SD* = 2.10; between 7 and 13 years of education attained, *M* = 9.26, *SD* = 2.21; IQ: between 81 and 123, *M* = 96.46, *SD* = 12.97).

The three groups were comparable in terms of age (Pearson’s chi-square test: *χ2* (4) = 2.82, *p* = .58), years of education (*χ2* (4) = 3.84, *p* = .42) and IQ (*χ2* (6) = 12.07, *p* = .06).

The two autistic adolescents groups (ASD TH and ASD FtF) were recruited based on the following inclusion criteria: (a) diagnosis of verbally fluent ASD certified by qualified clinicians using the DSM-5 guidelines (4) (b) age between 12-18 years; (c) native Italian speakers; (d) basic language abilities assessed by the language comprehension subtask of the BVN 12-18 (99), namely the Token Test (100). Exclusion criteria were: (e) previous history of brain injury or neurological disorder; (f) concurrent participation in Applied Behavior Analysis (ABA) or other communicative rehabilitation programs. The typical development control group (CG FtF) was selected according to criteria (b), (c) and (e).

All participants were assessed with the Raven Progressive Matrices (Italian norms by 101) before administering the material for the pragmatic assessment.

The groups were pseudo-randomized according to the family schedule and the organization of the timing of the assessment. Before participating in the research, both the participants and their caregivers gave written informed consent. The study was approved by the Bioethics Committee of University of Turin, protocol no. 0088488.

### 2.2 Material

#### 2.2.1 The assessment battery for communication

Participants’ communicative-pragmatic abilities were assessed with the equivalent Form A of the Assessment Battery for Communication (ABaCo), a validated clinical tool that assesses various aspects of pragmatic ability (45, 46, 83). The equivalent forms of the ABaCo consist of 68 items, divided into 4 scales: (1) Linguistic, (2) Extralinguistic, (3) Paralinguistic and (4) Context scales — each scale assessing both comprehension and production skills. For more details see the **Supplementary Material**. The equivalent forms of ABaCo have demonstrated excellent psychometric properties, showing high internal consistency (Form A, total score: α = .92). A more detailed overview of the psychometric properties of the ABaCo equivalent forms can be found in Bosco et al. (46).

The ABaCo battery consists of short video clip scenes (20–25s each), followed by specific semi-structured open questions and *vis-à-vis* interactions between the participant and the evaluator. In the comprehension tasks, the examiner asks the participant specific questions to see if he/she understood the protagonist’s communicative act. In the production tasks, the examiner asks the participant to complete an interaction.

##### 2.2.1.1 E-ABaCo- telehealth adaptation

In present study we created an adapted version of the ABaCo (form A; 46) for telehealth assessments and we called it e-ABaCo. The adaptation process involved three steps:

1. Identification of items requiring any modification: First, we identified the items that were not suitable for online assessment. These included items that required the experimenter and participant to manipulate physical objects in the room. For example, item L13 “Give me the pen” or item L11 “Can you give me that book?”. The other items we identified unsuitable for the online version were related to shared experiences or environmental observations, such as the weather, which were not applicable online because participants were in different locations and could not share the same experience. For example, item L1 “Today the weather is very nice” or item L7 “What do you like about this building?”.
2. Discussion among experts in pragmatics: A thorough discussion among experts in pragmatics was held for each item in order to plan its adaptation for use in the context of telehealth in the most ecologically valid way. For example, the item L7 “*What do you like about this building?”* was discussed in order to ensure contextual consistency and at the same time make the item more suitable for telepractice. It was adapted to “*What do you like about this season?*”. See **Table 1** for the full list of adapted items. The process involved only a limited number of items, which did not change the essential content of the items with respect to the original version.
3. Items’ modification: The final changes were applied to the items selected in step (1). A total of 8 items underwent modifications, out of 68, thus corresponding to 11.76% of the total items. A complete list of the original items and the new modified items can be found in **Table 1**.

**Table 1:** Items modified for the telehealth administration of ABaCo (Form A).

Scale	Subscale	Code	Original Item	Adapted item
Linguistic	Comprehension	L1	Today the weather is very nice.	Your hair is very short.
		L7	What do you like about this building?	What do you like about this season?
		L11	Can you give me that book?	Can you show me the palm of your hand?
		L13	Take the pen.	Touch the computer's webcam/camera.
		L16	Show me where the window is.	Close and open your eyes.
	Production	L38	Ask me for a tissue.	Ask me to touch my ear.
Extralinguistic	Production	X29	Tell me that there is a bad smell in here.	Tell me that there is a bad smell in the room you are in.
Paralinguistic	Production	P32	Order me to give you the pen.	Order me to take a pen.

### 2.3 Procedure

The autistic groups (ASD TH and ASD FtF) were recruited in collaboration with two rehabilitation centers: the Centro Riabilitazione Ferrero (Alba, Italy) and the Centro Autismo e Sindrome di Asperger (Mondovì, Italy). The participants with typical development (CG FtF), were recruited by advertising the study with flyers on the research group’s website and on social media platforms (Facebook, Instagram, X) and through personal contacts.

Participants in the face-to-face modality (ASD FtF and CG FtF) were tested in a quiet room; video clips were presented on a laptop while the experimenter and participants sat at a table facing each other. Participants in the telehealth modality (ASD TH) were tested in a quiet room in their home, via a remote participation via a free videoconferencing platform (i.e. Google Meet); the video clips were shared on screen with the participants after ensuring that the internet connection was stable and that the participants were clearly visible, with their face properly framed and optimally lit, to accurately assess the extra-linguistic and paralinguistic cues. To ensure an ecological environment, the experimenter remained visible to the participant throughout all the test session, with the camera switched on. In the event of technical problems, the examiner conducted a telephone consultation with the participant and/or their caregiver to resolve the issue.

Each session lasted approximately 45 minutes, regardless of the type of assessment, and was videorecorded with the consent of the participants and their caregivers. This allowed for offline scoring by reviewing the sessions administered by an independent, blinded rater. Participants’ responses to the ABaCo/e-ABaCo were scored on dedicated scoring sheets. For each task, a score of 0 (incorrect response) or 1 (correct response) was assigned based on the coding manual (45).

### 2.4 Data analysis

The analyses were conducted with IBM SPSS Statistics 29. Given the sample size composed by 3 small (N=15) subgroups, we opted for non-parametric tests, i.e. –one-way Kruskal-Wallis and Mann–Whitney *U* tests. The Kruskal–Wallis test examines the medians values of three or more independent groups to determine whether they originate from the same or different populations (102, 103). The Mann–Whitney *U* test, is a nonparametric method for comparing two independent groups based on their rank distributions (103, 104). In this study, these tests were used to assess the sensitivity of (e-)ABaCo in discriminating groups based on their pragmatic scores. Specifically, we examined whether (e-)ABaCo can discriminate pragmatic difficulties in adolescents on the autism spectrum (ASD FtF and ASD TH) compared to typically developing peers (CG FtF). In addition, via *ad hoc* pairwise comparisons, we examined the equivalence of (e-)ABaCo scores across the administration modalities by comparing the performance of ASD participants assessed face-to-face (ASD FtF) and via telehealth (ASD TH).

Thus, we conducted a series of Kruskal–Wallis test with group (ASD TH, ASD FtF, and CG FtF) as between-subjects and overall pragmatic performance (ABaCo total score), comprehension and production modality, and then for differences on the (e-)ABaCo scales (linguistic, extra-linguistic, paralinguistic, and contextual skills) as within-subjects factors. *Post-hoc* comparisons of group means were performed using the Mann-Whitney *U* test to assess differences in pragmatic ability, with adjustments made using the Bonferroni correction.

## 3 Results

### 3.1 (e-)ABaCo total score

**Figure 1:**
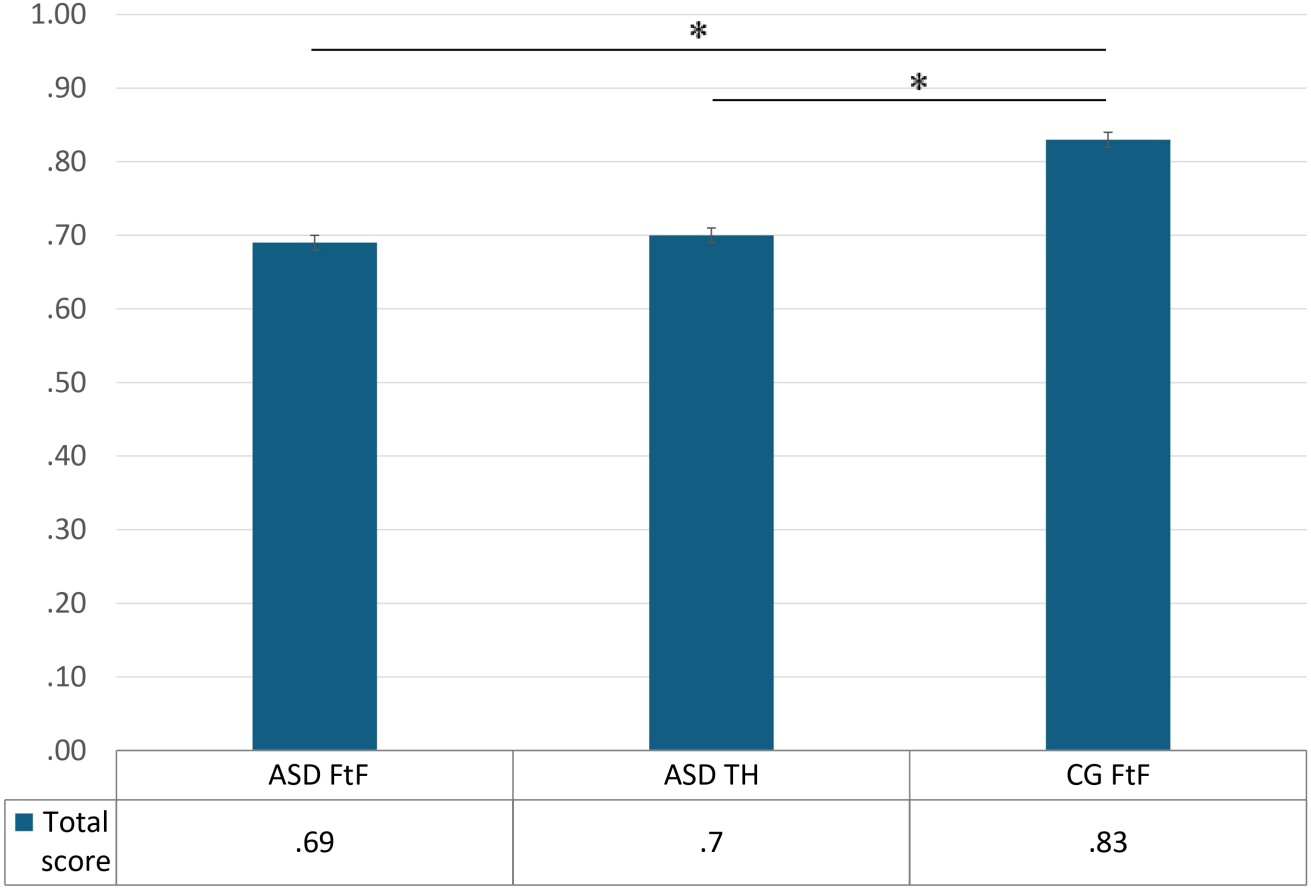
ABaCo mean total scores for each group examined: ASD FtF (ASD face-to-face), ASD TH (ASD telehealth) and CG FtF (Control Group face-to-face). Error bars indicate standard errors of the mean. Statistical significance (*p* < .05) is marked with an asterisk (*).

A Kruskal–Wallis test shows that there was a significant main effect of group — ASD TH, ASD FtF and CG FtF — on the global ABaCo score *H*(2) = 20.08, *p* < .001. A pairwise *post-hoc* comparison (Bonferroni correction) shows that the CG FtF performed significantly better than the ASD FtF (*p* < .001) and the ASD TH group (*p* < .001). As hypothesized, no difference was found between the performance of ASD in face-to-face vs. ASD in telehealth group (*p* = .54) (see **Figure 1**).

### 3.2 (e-)ABaCo comprehension and production

Looking more in detail to comprehension and production composite scores, a Kruskal-Wallis test showed a significant effect of group on (e-)ABaCo comprehension scores, *H*(2) = 14.36, *p* <.001, and (e-)ABaCo production scores, *H*(2) = 13.10, *p* = .001. The pairwise *post-hoc* comparisons (Bonferroni correction) showed, in line with previous results, that the non-clinical control group CG FtF scored significantly higher than the ASD face-to-face group on both comprehension (*p* = .004) and production (*p* < .001) skills. In addition, as expected, the CG FtF outperformed the ASD telehealth group on both comprehension (*p* < .001) and production (*p* = .01).

**Figure 2:**
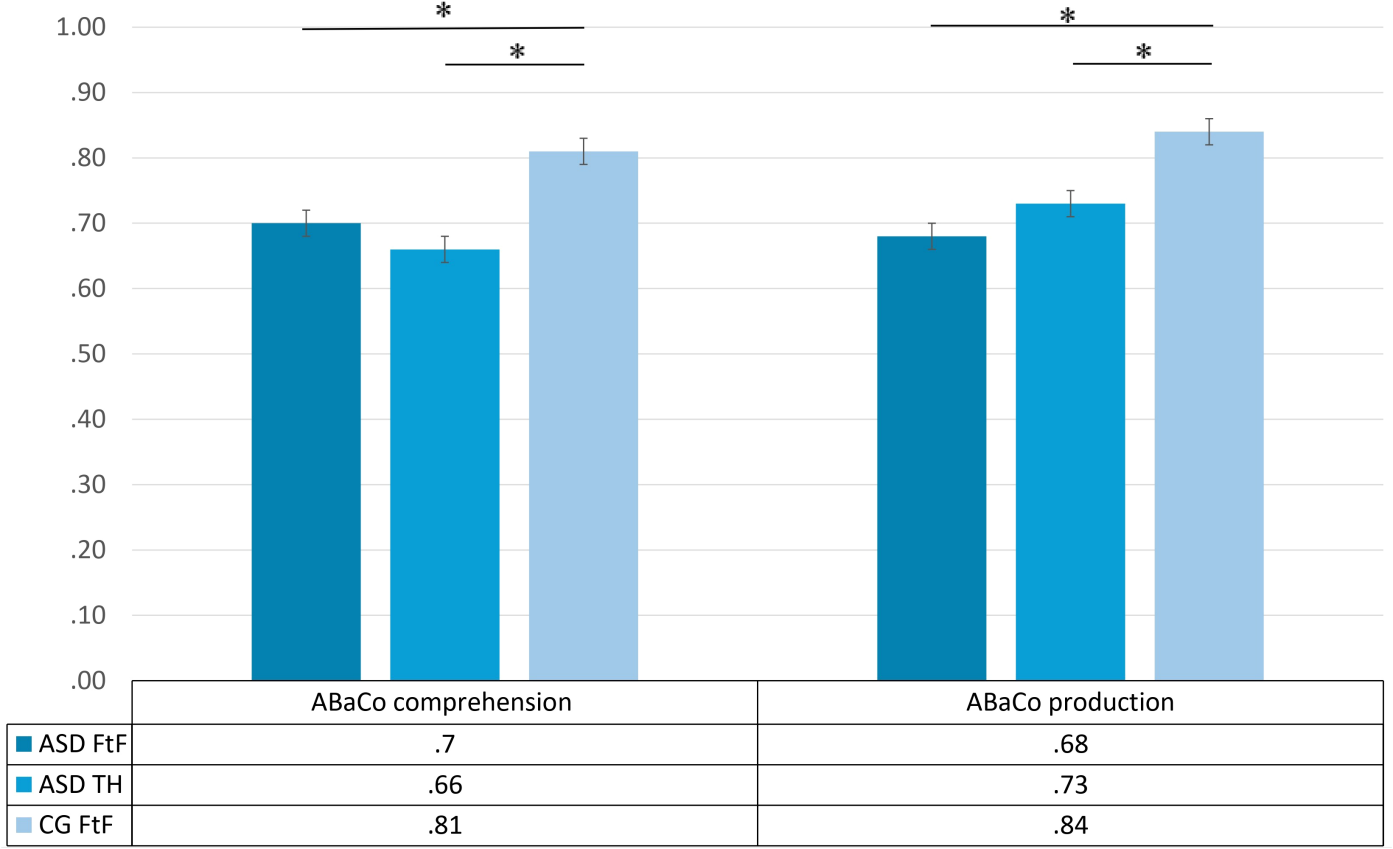
ABaCo comprehension & production scores for each group examined ASD FtF (ASD face-to-face), ASD TH (ASD telehealth) and CG FtF (Control Group face-to-face). Error bars indicate standard errors of the mean. Statistical significance (*p* < .05) is marked with an asterisk (*).

Finally, as expected, no significant differences were found between the ASD face-to-face group and the ASD telehealth groups for either comprehension (*p* = .49) or production abilities (*p* = .34) (see **Figure 2**).

### 3.3 (e-)ABaCo scales

**Figure 3:**
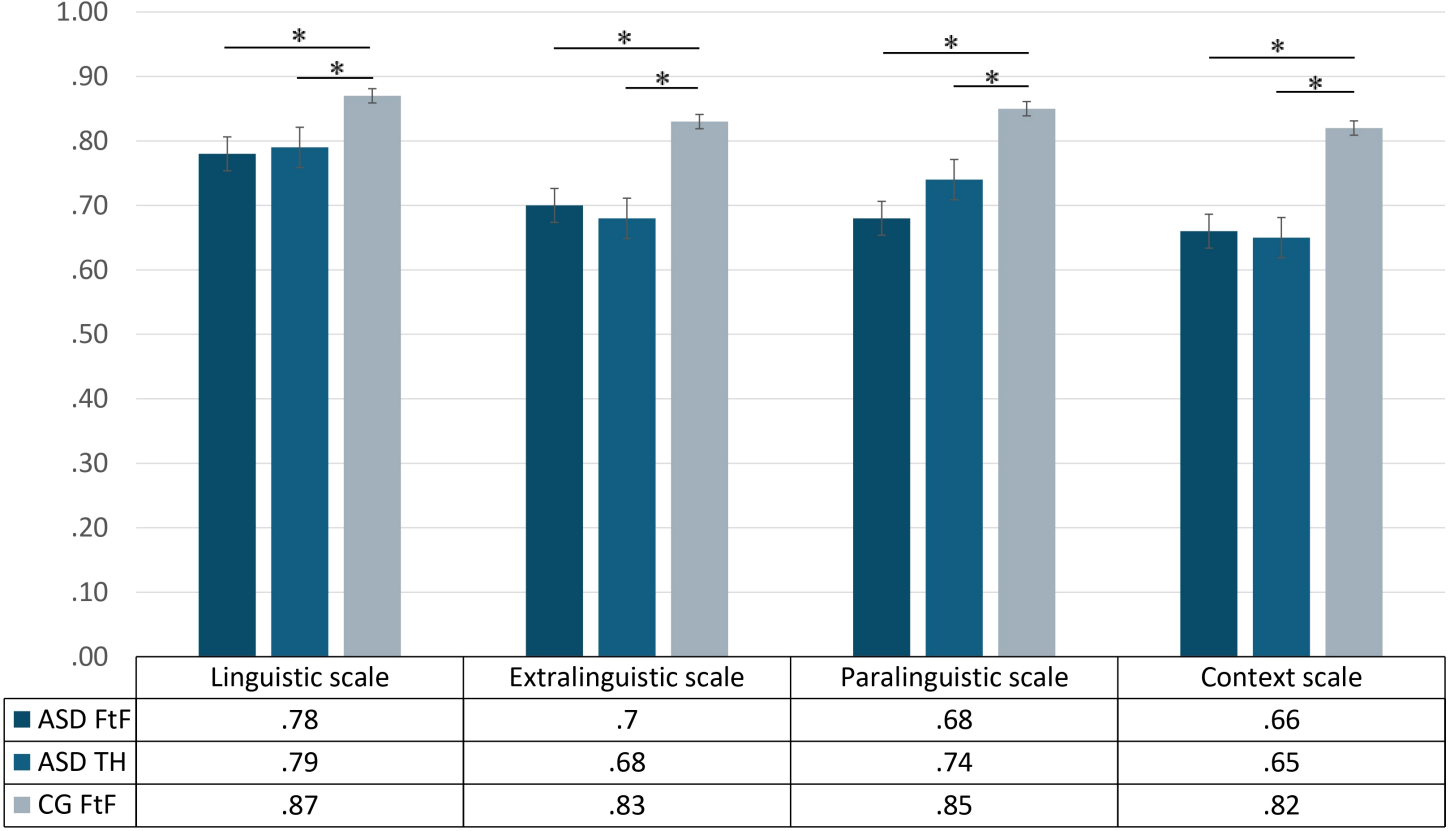
ABaCo scales scores for each group examined ASD FtF (ASD face-to-face), ASD TH (ASD telehealth) and CG FtF (Control Group face-to-face). Error bars indicate standard errors of the mean. Statistical significance (*p* < .05) is marked with an asterisk (*).

The Kruskal-Wallis test revealed significant group differences in all (e-)ABaCo scales: linguistic (*H*(2) = 11.77, *p* = .003), extralinguistic (*H*(2) = 16.13, *p* < .001), paralinguistic (*H*(2) = 11.87, *p* = .003) and context (*H*(2) = 6.84, *p* = .03), see **Figure 3**. Pairwise *post-hoc* comparisons (Bonferroni) showed that the CG FtF scored significantly higher than the ASD face-to-face group on all scales, including linguistic (*p* = .001), extralinguistic (*p* = .001), paralinguistic (*p* < .001) and context (*p* = .02). Similarly, the CG FtF performed better than the ASD telehealth group on all scales: linguistic (*p* = .01), extralinguistic (*p* < .001), paralinguistic (*p* = .02) and context (*p* = .02).

In line with the expectation, no significant differences were found between the ASD face-to-face and ASD-telehealth groups in any of the scales analyzed, with comparable performance observed in the two groups for linguistic (*p* = .51), extralinguistic (*p* = .63), paralinguistic (*p* = .24) and context (*p* = .89).

## 4 Discussion

The aim of this study was to present the adaptation and evaluation of the feasibility of ABaCo, Form A (45, 83), in telehealth (e-ABaCo), as well as to verify its sensitivity to assess the pragmatic profile of autistic adolescents in telehealth compared to those of ABaCo in face-to-face modality. To this end, we analyzed the performance of three different groups: ASD FtF, ASD TH and CG FtF group. The study examined different forms of expression, including linguistic, extra-linguistic (such as gestures and facial expressions) and paralinguistic elements (such as prosody), as well as sensitivity to social context and, finally, composite scores reflecting comprehension and production abilities across the above-mentioned modalities.

A novelty of this study was the investigation of telehealth as a methodological approach. Telehealth has gained considerable relevance in recent years as a valid alternative for conducting assessments in various areas such as diagnostic, psychological and neuropsychological assessments, intervention programs, and educational counseling (50, 74, 80, 81). However, there is only a limited number of tools specifically developed, in the Italian context, for the pragmatic assessment in telehealth (82) and so far, to our knowledge, there is no tool specifically developed for remote assessment of pragmatic skills in adolescence in Italian language and, even more specifically, in the autism spectrum. For this reason, demonstrating the sensitivity of the e-ABaCo would be valuable for both research and clinical practice.

Consistently with our hypotheses, results showed no significant differences in communicative-pragmatic ability between autistic adolescents assessed face-to-face (ASD FtF) and via telehealth (ASD TH) in all pragmatic aspects examined. These include the pragmatic total score, comprehension and production composite scores and the four scales (linguistic, extra-linguistic, paralinguistic and context). These results provide evidence of equivalence between the administration of e-ABaCo and ABaCo, and show the effectiveness of e-ABaCo in discriminating between ASD and CG samples. More in detail, according to the second aim, the CG FtF group, which consisted of typically development adolescents, performed significantly better than the two ASD groups (FtF and TH) in all pragmatic aspects examined, including pragmatic total score, comprehension and production skills, and all the expressive means examined, emphasizing the sensitivity of our tool in discriminating between clinical and non-clinical populations based on their pragmatic performance, thus confirming data in the literature regarding the possible presence of various difficulties at pragmatic level in the autism spectrum.

These data are consistent with results showing a substantial equivalence between tele-assessments and face-to-face modalities for tasks investigating language in different clinical groups (80, 105), such as adults and elderly with acquired language impairment (106, 107), individuals with cognitive impairment (108, 109), and with psychiatric disorders (110). The same analogy applies to the effectiveness of the tele-assessment of cognitive skills (MMSE; 111–113) via neuropsychological tools such as the Mini‐Mental State Examination. All these studies found similarities between tools used in a face-to-face setting and those adapted for the use in telehealth, particularly for synchronous tests requiring real-time interaction between examiner and participant (80).

The present results are in line also with the few available data concerning autism (Boisvert et al. (114) and Sutherland et al. (66), which highlight that remote assessment and telerehabilitation interventions are potentially equivalent to face-to-face services (for a review see 81).

The results of the present study are also consistent with the demonstrated equivalence between the APACS Brief Remote and the face-to-face version of APACS, as participants consistently achieved similar levels of performance in both formats (82). Both the e-ABaCo and the APACS Brief Remote are designed to assess pragmatic ability; however, they target different and only partially overlapping pragmatic phenomena. Indeed, unlike the APACS Brief Remote, the e-ABaCo offers a comprehensive assessment that also includes non-verbal/extralinguistic and paralinguistic means of expression, essential components for a full understanding of the pragmatic profile of each individual. Moreover, while Bischetti et al. (82) focused on validating a brief version of the APACS in a remote setting with healthy adults, our study refers to autistic adolescents. Finally, an important methodological decision in our study was to conduct the assessment exclusively on a computer instead of opting for both a computer and a smartphone/tablet. Bischetti et al. (82), indeed, found a difference in perceived difficulty between people who used a smartphone and those who used a computer. This could be due to the distraction of pop-up notifications when using the smartphone or other important factors such as the size of the screen, while computer use was more reflective of face-to-face experiences (51, 115). These aspects are specifically important since controlling for this variable can assure high methodological rigor, especially in the assessment of non-verbal components.

In a broader perspective, the results are consistent with data in the literature (10, 20, 21, 29, 116, 117) indicating lower performance (in face-to-face modality) of autistic adolescents, autistic adults and adults with subclinical features compared to non-autistic participants on the social-pragmatic inference tasks. In Angeleri et al. (21), difficulties were found in children and autistic adolescents in both comprehension and production skills in linguistic and extra-linguistic domains, as well as in the paralinguistic and social aspects of communication, assessed with the four scales of ABaCo. New findings show that not only the autistic group, but also the subclinical group, i.e. adults with autistic traits but without an ASD diagnosis, differ from the comparison group in terms of more incongruent meaning-related inferences (10) and fewer production narratives focused on the main topic of the conversation (118).

From a macro perspective, this study shows that telehealth is a promising alternative method and an important resource for overcoming geographical barriers and improving access to healthcare. In particular, it helps to ensure that individuals who would otherwise face barriers, such as residents of rural areas, patients with limited transportation options, and those who are homebound, are highly engaged. This is particularly true of the challenges encountered in services and diagnostic procedures for ASD (60). The existing literature on the effectiveness of telehealth for people on the autism spectrum shows mixed results (74, 119). While telehealth services are generally well received by autistic youth and their caregivers, technical issues, such as the lost connection, remain a significant barrier to effective service delivery (119). In addition, the accessibility of telehealth may be limited for people with socioeconomic or technical-geographic disadvantages (120). Furthermore, not all people are able to use technology, and for certain populations, such as older adults or those with limited digital literacy, it can be a significant barrier to access and participation. Despite these challenges, telehealth remains a viable alternative for autistic people when in-person services are not possible. It provides opportunities for therapeutic interventions, family counseling, teaching skills, maintaining skills, and addressing behavioral and communication challenges.

In particular, e-ABaCo appears to be a viable and effective approach to address the communicative-pragmatic challenges of autistic adolescents in telehealth. For the first time in telepractice, it was possible to draw a profile of communicative pragmatic difficulties and strengths of a group of autistic adolescents.

The study raises relevant points and opens to several future lines of research, but it is not exempt from limitations. One limitation may be referred to a rather small sample size and to the low number of female participants. However, the sex imbalance can be attributed to the higher prevalence of ASD in men compared to women, with approximately 70% of diagnosed cases being male (121). As this is a first study with e-ABaCo, the results provide a promising basis for further studies with larger samples, which could help to confirm and strengthen the results using parametric statistical tests and sensitivity analysis by different perspectives (e.g. ROC analysis or classification models) and assess the effects of gender or other demographic variables such as education level and age. Another aspect that could be investigated more in detail is the extent to which technological mediation influences certain dynamics, such as non-verbal communication, the environment between assessor and participants and emergency management (122).

The use of one equivalent form of ABaCo will allow future assessment to be conducted for longitudinal monitoring of changes in pragmatic skills, e.g., in pre-post evaluations of intervention effectiveness or follow-up, while minimizing the influence of practice effects. Upcoming/future studies will be focused on these aspects. Future research could, indeed, adapt for telehealth the other equivalent form of ABaCo, form B, and thus further expand the potential for reliable longitudinal studies in telehealth. The possibility to monitor the pragmatic performance over time is particularly important given the evidence that pragmatic abilities can fluctuate over the course of the disorder (see 29, 123, 124), and given the prevalence of pragmatic impairment in a number of clinical populations, such as individuals with schizophrenia (93, 125–127), traumatic brain injury (89, 128), children with special needs (88), and no clinical population such as in healthy aging (129).

In summary, these results support the conclusion that the e-ABaCo is a reliable tool for conducting an effective assessment that not only shows equivalence with the original face-to-face version, but also proves to be effective in identifying variabilities within a clinical group (ASD) in a telehealth setting in an Italian-speaking population. These data contribute to fill the gap in the literature, as research on pragmatic tele-assessments in adolescence and in autism is limited. We believe that this study, which demonstrates the effectiveness of e-ABaCo, will help to improve both the accessibility and efficiency of the assessment in telehealth, as well as to promote the integration of pragmatic assessment into the routine assessment and the monitoring of the fluctuation of linguistic and communicative skills.

## Data Availability

The original contributions presented in the study are included in the article/**Supplementary Material**. Further inquiries can be directed to the corresponding author.

## Footnotes

1 We opt to use identity-first terminology commonly preferred by autistic people/people on the autism spectrum (see e.g.,^8^, ^9^).
