# Supplementary material for: Assessing communicative-pragmatic ability in telehealth: e-ABaCo in autistic individuals
Source: Front Psychiatry. 2025 Apr 28;16:1568108. doi: 10.3389/fpsyt.2025.1568108 (PMC12066485; doi:10.3389/fpsyt.2025.1568108)
Supplement: Supplementary file 1 [file DataSheet1.pdf]

Supplementary material

The following table offers a detailed description of each scale of the equivalent forms of the Assessment Battery for Communication (ABaCo, Bosco et al., 2012)

SCALE	SUBSCALE	DESCRIPTION	N. ITEMS
LINGUISTIC	• *Comprehension*	Assesses comprehension of basic communication acts (BSA, assertions, questions, requests, commands, Kasher, 1981)[1]. For direct and indirect communicative acts (Searle, 1975), irony and deceit the examiner shows the participants a short video clip in which an actor asks a partner a question and the partner answers.<br><br>Participants are asked to demonstrate their understanding of the communicative interaction by answering specific questions about the scene. In this scale, the communicative exchange in the scene is linguistic.	14
	• *Production*	Assesses the production of BSA (statement, a question, a request, or a command). For direct and indirect communicative acts, irony and deceit participants the examiner shows the participants a video clip where two interlocutors are communicating verbally: an actor ask to a question to her partner. The participant is asked to respond to video scenarios verbally.	14
EXTRA-LINGUISTIC	• *Comprehension*	Assesses comprehension of BSA — assertions, questions, requests or commands — direct and indirect communicative acts, irony and deceit using only gestures. In this scale, participants are asked to see short video clips in which actors communicate through gestures only. The participant is required to demonstrate their understanding of the communicative act expressed by gestures by answering specific questions about the scene.	10
	• *Production*	Assesses comprehension of BSA — assertions, questions, requests or commands — direct and indirect communicative acts, irony and deceit. In this scale, participants watch video clips in which the actor asks a question using gestures and they are asked to respond with gestures as well.	13
PARALINGUISTIC	• *Comprehension*	The examiner shows the participants video clips for assessing (1) BSA (assertions, questions, requests, commands) in which the actor in the scene speaks an invented language. The examiner provides four	6

[1] According to Kasher (1981), basic communicative acts (BSA) such as assertions, questions, requests and commands are the simplest linguistic acts that can be understood and produced.

		verbal response options; (2) Recognition of basic emotions (e.g. anger, sadness, sadness, fear) in which actors in the scene expressed through paralinguistic cues. The examiner provides four verbal response options; (3) Paralinguistic contradiction in which the actors in the scene express verbal content that contradicts the accompanying paralinguistic cues. For example, 'I like it very much' with a tone that suggests the opposite.	
	• *Production*	The examiner asks the participant to perform basic speech acts (i.e. to formulate assertions, questions, requests and commands), paying particular attention to paralinguistic cues. The examiner indicates the semantic content of the communicative act (e.g. 'Ask me if it is sunny today'). In addition, this scale assesses communicative acts that convey an emotion or mood. In this case, the examiner provides both the semantic content and the emotion to be expressed (e.g. 'Tell me that you have received a letter — say it in a happy way') and the participant has to perform communicative acts that convey the emotion or mood indicated.	6
CONTEXTUAL	• *Comprehension*	Assesses comprehension of the adequacy or inadequacy of communicative acts in relation to discourse norms (Grice, 1975) and social norms. Participants are shown short video clips in which either discourse norms or social norms are respected or violated. Violations of discourse norms follow the violation of a Grice’s maxim (Grice, 1975) and may involve the partner in the scene giving a general, false, irrelevant or ambiguous answer; violations of social norms may involve responses that are inappropriate for the given context, e.g. to the question 'Could you lend me your pen?' the partner might respond impolitely and say 'I don’t want to be disturbed!"	3
	• *Production*	The examiner asks the participant to produce communicative acts at different levels of formality or informality, providing the semantic content of each required communicative act.	2
	Total		68
